# Prospective evaluation of locoregional control in oral cavity squamous cell carcinoma with lower or mid infratemporal fossa involvement treated with surgery and adjuvant concurrent chemoradiotherapy

**DOI:** 10.3332/ecancer.2026.2098

**Published:** 2026-03-19

**Authors:** Prasoon Mishra, Rahat Hadi, Ajeet Kumar Gandhi, Madhup Rastogi, Rohini Khurana, Ashish Singhal, Surendra Prasad Mishra, Anoop Srivastava, Avinav Bharati, Ashish Chandra Agarwal, Avinash Poojari, Vachaspati Kumar Mishra, Raunaq Puri, Akanksha Manral, Vikas Gupta, Bhoopendra Pratap Vishwaranjan, Saumyta Mishra

**Affiliations:** 1 Department of Radiation Oncology, Dr. Ram Manohar Lohia Institute of Medical Sciences, Lucknow 226010, India; 2 Department of Surgical Oncology, Dr. Ram Manohar Lohia Institute of Medical Sciences, Lucknow 226010, India; 3 Department of Otorhinolaryngology, Dr. Ram Manohar Lohia Institute of Medical Sciences, Lucknow 226010, India; 4 Department of Surgical Oncology, Mahavir Cancer Sansthan, Patna 801505, India

**Keywords:** low and mid infratemporal fossa involvement, ITF clearance, concurrent chemoradiotherapy, loco-regional control, locally advanced OCSCC

## Abstract

**Purpose:**

Oral cavity squamous cell carcinoma (OCSCC) is the most prevalent malignancy of the head and neck malignancy in India. In locally advanced OCSCC, a combination of definitive surgery followed by postoperative radiotherapy (RT), with or without concurrent chemotherapy, offers superior oncologic outcomes compared to definitive chemoradiotherapy alone. The prognosis of T4b OCSCC with infratemporal fossa (ITF) involvement largely depended on the degree of invasion. Tumours confined to the lower or mid ITF tend to have relatively better outcomes than those with extensive or high-level involvement. This study prospectively evaluates the treatment outcomes in patients with OCSCC involving the lower or mid ITF who underwent curative surgical resection followed by adjuvant concurrent chemoradiotherapy (CCRT).

**Methods:**

This is a single-arm prospective interventional study between January 2021 and October 2022, which included 20 patients of clinically T4b OCSCC as per the American Joint Committee on Cancer (8th edition) with lower or mid ITF involvement. Patients with tumour invasion into the skull base, carotid artery, prevertebral fascia or pterygomaxillary fissure were excluded to maintain a uniform study cohort. All patients were treated with curative intent with surgery include ITF clearance followed by adjuvant CCRT. Primary endpoint was to evaluate loco-regional control (LRC) and secondary end point was to assess acute toxicities according to the Common Terminology Criteria for Adverse Events v5.0. Kaplan-Meier survival analysis was done for assessing disease free survival (DFS), overall survival (OS).

**Results:**

Median follow-up was 11.5 months (4–21 months). The median age was 39 years (range 32–67) with 95% males. Buccal mucosa was most common site with 19 patients (95%). All patients were clinically cT4b (Stage IV) stage involving low/mid ITF involvement with low ITF were 15(75%) and mid ITF patients were 5(25%) patients. All patients underwent definitive ITF clearance and received adjuvant CCRT with weekly concurrent cisplatin 35–40 mg/m^2^ to a median number of 6 cycles (5–7 cycles). Median RT dose was 64 Gray (60–66). 08 (40%) patients had grade 3 oral mucositis and odynophagia each. Skin toxicity and oral pain was present in 6(30%) and 5(25%) patients, respectively. Grade 4 toxicities were not observed. LRC at 3 months was 73.68% with 4 local and 1 locoregional failures only. LRC at 1 year was 36.8% with 8 local, 2 local-distant, 1 loco-regional-distant and 1 distant failure only. Median DFS was 10.4 months and median OS was not reached; four patients died. LRC at 3 months was better for patients with low ITF disease as compared to mid ITF disease (85.7% versus 40% (*p* = 0.04) and at 1 year was 42.8% versus 40% (*p* = 0.50).

**Conclusion:**

Locally advanced OCSCC with low ITF involvement may benefit from tri-modality therapy of surgery with ITF clearance followed by adjuvant CCRT. The acute toxicity observed in study was within acceptable limits.

## Background

In India, head and neck cancers represent major health issue, contribute nearly 30% of all new cancer case in which lip and oral cavity constitute approximately 140,700 new cases. Oral cavity carcinoma is the most common subtype among males, mainly linked to the widespread use of tobacco in multiple forms. Its incidence is approximately 10.3% and it is a 2nd most common leading cause of cancer deaths (8.7%) [1, 2]. Over 50% present with advanced-stage disease, with gingiva-buccal sulcus complex cancers being the most prevalent [2].

Surgery is the primary treatment for oral cavity squamous cell carcinoma (OCSCC), with adjuvant radiotherapy (RT) with or without chemotherapy recommended for high-risk features such as advanced stage, positive margins or perineural and lymphovascular invasion (LVI). The optimal RT dose, fractionation and concurrent chemotherapy remain complex and are addressed through risk stratification frameworks [3–5]. The EORTC 22931 and RTOG 9501 trials established the foundation for the risk-based selection of adjuvant RT and concurrent chemoradiotherapy (CCRT) in postoperative OCSCC [6, 7].

These studies identified an intermediate-risk group between low-risk patients, who may not need adjuvant therapy and high-risk patients requiring adjuvant CCRT. This group includes pT1-4 tumours with close margins (1–5 mm), perineural invasion (PNI), LVI, involved nodes or poor differentiation.

Head and neck surgeons faces challenges in locally advanced OCSCC especially when the disease involves the infratemporal fossa (ITF) and are associated with a median survival of approximately 6 to 9 months [2, 8]. Advanced oral cavity cancers with ITF involvement are classified as T4b disease and designated as ‘very advanced disease’ in American Joint Committee on Cancer (AJCC) 8th edition staging system [9].

The **ITF** is a structure situated deep to the mandibular ramus and below the zygomatic arch. It contains the **pterygoid muscles**, **V3**, **chorda tympani**, **otic ganglion**, **maxillary artery** and **pterygoid venous plexus**. The **masseter muscle** is typically excluded [10–13].

ITF involvement is described as category **low**, **intermediate** or **high** based on anatomical spread, from local muscle involvement to extension into the intracranial compartment. Low and intermediate are operable with good outcomes, while high ITF, has variable prognosis [14–16].

The anatomy of the ITF and masticator space (MS) complicates cancer evaluation. Radiological assessment is challenging, and trismus limits clinical examination [17–19]. Due to neurovascular structure in the MS complete resection of tumour is very difficult.

cT4b oral cavity cancers are traditionally inoperable and managed palliative with radiation and chemotherapy or both [20, 21]. Due to complex anatomy ITF tumours were rarely resected but recent studies show improved outcomes with compartment resection especially below the mandibular sigmoid notch [15, 22–26]. In few studies neoadjuvant chemotherapy (NACT) has been used to downstage tumours and improve resectability [27–29].

cT4b carcinoma of oral cavity has poor outcomes. Patient with minimal involvement of ITF (mid-lower 1/3) may have better outcome as compared to other cT4 OCSCC patient if treated aggressively with surgery and adjuvant CCRT [10].

We aim to evaluate patients of OCSCC with minimal ITF (mid-lower 1/3) involvement treated with surgery followed by adjuvant CCRT administered as per our institutional protocol in view of the aggressive nature of ITF involvement in terms of loco-regional control (LRC) and acute toxicity of treatment.

## Methods

### Study design

20 patients of cT4 OCSCC (as per AJCC 8^th^ edition) with low or mid ITF involvement were included in this single arm prospective interventional study from January 2021 to October 2022. All patients underwent curative surgical resection with standardized ITF clearance followed by**,** adjuvant CCRT was administered to all patients. No patients in this cohort received NACT prior to surgery.

The study was approved by the institutional ethics committee (IEC No.166/20/RMLIMS/ 2020) and written informed consent was taken from all patients prior to the enrolment.

Eligible patients had OCSCCs (except lip), aged 18–80 years, of either sex with a Karnofsky performance score ≥70, ITF Involvement mid and lower defined on cross-sectional imaging (contrast-enhanced CT or MRI) as Low ITF involvement (infranotch) on imaging as tumour localized below the mandibular (sigmoid) notch, structures involving such as the medial pterygoid, masseter or retroantral fat and Mid ITF involvement (supranotch) was defined as tumour extending superiorly above the notch with involvement of lateral pterygoid, temporalis insertion or upper pterygoid plates, without involving skull base or intracranial extension [15, 16, 30],with pathologically complete and appropriate surgical resection of primary disease and nodes with 1 or more of the following risk factors for adjuvant treatment: pT3/pT4, ≥pN2a, close margin (1–5 mm), PNI, lymphovasular invasion and depth of invasion (> 4 mm for tongue and ≥7 mm for buccal mucosa) [4, 31], high grade tumour, adequate bone marrow reserve, non metastatic disease, as per institutional protocol, ITF involvement itself was considered a high-risk feature; therefore, all patients were planned for adjuvant CCRT irrespective of classical high-risk factors such as margin positivity or extra capsular extension as stated in landmark study (category 1 as per NCCN) [6, 7].

Excluded patients were non-squamous cell carcinoma histology, high ITF involvement extending to the temporal fossa or infiltration into the pterygomaxillary fissure, carotid artery encasement or prevertebral fascia. Cases of T4b disease except for isolated ITF involvement were also excluded. Additional exclusions included patients with synchronous or metachronous second malignancies, metastatic disease, pregnancy, lactation or active infections such as HIV, Hepatitis B or Hepatitis C. Patients indicated for chemo-radiation but deemed unfit due to co morbidities, poor general condition or unwillingness to undergo chemotherapy were also excluded from the study.

### Clinical evaluation

All patients were staged in accordance with the criteria outlined in the 8th edition of the AJCC Cancer Staging Manual, published in 2017. A thorough medical history and physical examination were conducted, along with routine hematological, renal and hepatic function tests, post-operative histopathological reporting, necessary CECT/MRI of the face and neck, chest X-ray (P/A view), abdominal ultrasonography and glomerular filteration rate assessment when clinically indicated.

***Surgery***

All patients underwent a standardized surgical procedure for ITF compartmental clearance, involving bite composite resection with comprehensive neck dissection (modified neck dissection type II/III based on nodal burden). Complete clearance was achieved by removing all muscles, fibro-fatty tissue, neurovascular bundles, retroantral fat and pterygoid plates within the specified region, adhering to the compartment surgery concept of removing the whole anatomical subunit encompasses the disease. Removal of the buccal branches of the facial nerve is required in advance diseases, if feasible upper division of the facial nerve and the marginal branch has to be preserved. Segmental mandibulectomy is commonly performed due to bone involvement or significant paramandibular spread, with reconstruction typically done using a bipaddled pectoralis major myocutaneous flap. All patients received adjuvant treatment as per surgical histopathology report [24].

Patients received oral hygiene counseling, dental prophylaxis with fluoride application, instructions for using oral rinses (benzydamine, sodium bicarbonate, saline gargles), clotrimazole lozenges during RT and their weight was recorded prior to treatment, with written informed consent. Gap between surgery and start of RT was kept ≤6 weeks.

### Radiotherapy

## Simulation and planning

CT simulation with contrast enhancement was performed using a 16-slice CT simulator (Siemens Somatom Sensation) with patients in a supine position; neck extended and immobilized using a carbon fiber base plate and a thermoplastic U-type extended face mask. Contrast enhanced images were acquired with a 3 mm slice thickness.

CT images were imported to the MONACO SIM (Version 5.11.03) contouring station via DICOM 3.0. Target volumes and organs at risk were contoured according to ICRU 50 & 62 guidelines. Clinical target volumes (CTVs) included the tumour bed and nodal regions, with a uniform 5 mm margin added to generate planning target volume, as per institutional protocol. The volumes taken have been presented in Table 1 [32, 33]. The data were then transferred to the XIO (Version 5.0) treatment planning system for further planning.

## Dose prescription

**Figure 1.**
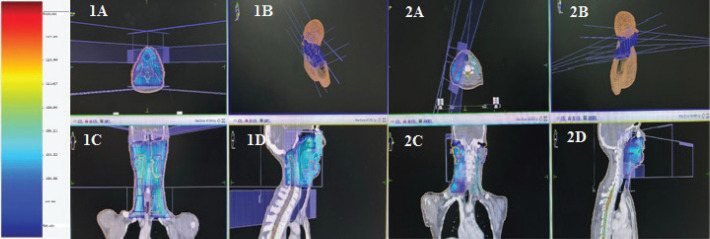
(1a–1d): Radiation treatment planning images of target volume delineation and dose distribution with parallel opposed lateral field matched with LAN. (2a–2d): Ipsilateral parallel oblique field.

RT was administered in two sequential phases, delivering a total dose of 60–66 Gray (Gy) in 30–33 fractions (2Gy per fraction), administered on weekdays (Monday through Friday) over a period of approximately 6 to 6.5 weeks. Patient was treated with three-dimensional conformal RTeither with parallel opposed anterior—posterior oblique field or bilateral parallel opposed lateral field matched with low anterior neck field (LAN) in 2 phases Figure 1 (1a–1d) via 6 MV photons along with concurrent chemotherapy with weekly Cisplatin 40 mg/m^2^. In the first phase, treatment was delivered using two parallel-opposed lateral photon fields matched with LAN field to encompass the bilateral face and neck including the lymphatic drainage areas to the dose of 44 Gy in 22 fractions. In the second phase, the cord is off by shifting the posterior border anteriorly and removing the LAN field followed by treating the target volume to the total dose of 60–66 Gy by bilateral parallel-opposed lateral fields. A representative dose colour wash distribution is illustrated in Figure 1. If clinically indicated and technically feasible, parallel opposed oblique fields were used to deliver dose up to 60–66 Gy to the Ipsilateral face and neck Figure 1 (2a–2d). Treatment plans were assessed using dose-volume histograms, planar and 3D isodose displays slice by slice and modified accordingly**.**

Organ-at-risk dose constraints included a spinal cord Dmax ≤ 45 Gy and PRV Cord Dmax ≤ 50 Gy. Position verification was done by Electronic Portal Imaging Device twice weekly in the first week and then once weekly. Treatment was administered using a source-to-axis distance technique with 6 MV X-ray beams from a Linear Accelerator (ELEKTA Infinity/Synergy, Crawley, UK) with a 1 cm collimator leaf width at the Isocentre.

Patients were assessed weekly during RT with body weight recorded before and during treatment using a standardized weighing machine. Acute radiation toxicities including oral mucositis (OM), skin toxicity, odynophagia, dysphagia, oral pain and hematological toxicities were graded per Common Terminology Criteria for Adverse Events v5.0 [34] from the start of RT to 90 days post-treatment. Oral hygiene, fungal infections and nutritional status were also assessed weekly.

## Post treatment follow up

Post-treatment completion, visits began 4 weeks after RT completion, followed by monthly visits for the first year and bimonthly for the second year. A minimum 6-month follow-up was conducted with documentation of toxicities**.** Locoregional status was evaluated at each visit of follow up with imaging (CECT/MRI/PET-CT) at 3 months after completion of RT to assess LRC. During subsequent follow-up, clinical examination was performed at every visit (monthly during the first year and bimonthly during the second year). Imaging was not performed at every visit but was repeated selectively at 3-month intervals in the first year when clinically indicated or earlier if new symptoms or suspicious findings were detected on clinical examination**.**

## Statistical analysis

Statistical analysis was done with Statistical Package for Social Sciences (v25). Quantitative data were summarized with median, mean and standard deviation. LRC was defined as the absence of local and/or regional failure Locoregional failure defined as biopsy proven disease in postoperative bed or cervical nodes or both**.** LRC rates were calculated using crude proportions and comparisons between groups were descriptive without formal statistical testing. Disease free survival was measured from date of surgery till date of locoregional failure, distant metastasis or disease-related death, while overall survival (OS) was calculated from date of registration to the date of death from any cause. Survival rates were calculated using Kaplan–Meier method. The patients who were alive at the time of data cut off time or lost to follow-up were censored for survival analysis.

## Results

Patient characteristics have been summarized in Table 2. Median follow-up was 11.5 months (4–21) months. All 20 Patients have completed the treatment. All patients underwent a standardized surgical procedure for ITF clearance with median number of lymph node dissected is 29 (16–61), only one patient with less than 18 nodes dissected. 17 patients had unilateral neck dissection (01 patients had supraomohyoid neck dissection, 19 had modified radical neck dissection and 1 had radical neck dissection) and 3 had bilateral neck dissection. 01 (05%) Patients had segmental mandibulectomy and 14 (70%) had hemi-mandibulectomy. 05 patients had upper alveolectomy. 12 out of 20 patients received unilateral RT and the remaining 08 received bilateral RT. All patients were pathologically pT4b (Stage IV) with ITF involvement. Median RT dose was 64 Gy (60–66 Gy). The median time interval between surgery and beginning of RT was 41 days (range: 30–49). The median overall treatment time defined as the interval from the date of surgery to the completion of postoperative RT was 107 days (75–123 days). The median duration of radiation therapy was 46 days (range: 35–52). The treatment compliance was good with all the patients receiving the full doses of RT as planned. All patients had infused concurrent chemotherapy with weekly cisplatin at a dose of 35–40 mg/m^2^, administered for a median of six cycles (range: 5–7). Out of 20 patients 9 patients (45%) received a reduced dose of 35 mg/m^2^ per week due to borderline renal function or prior grade 2 hematological toxicities. Dose reductions were made as per institutional safety protocols to maintain tolerability.

### Toxicity analysis

**Figure 3.**
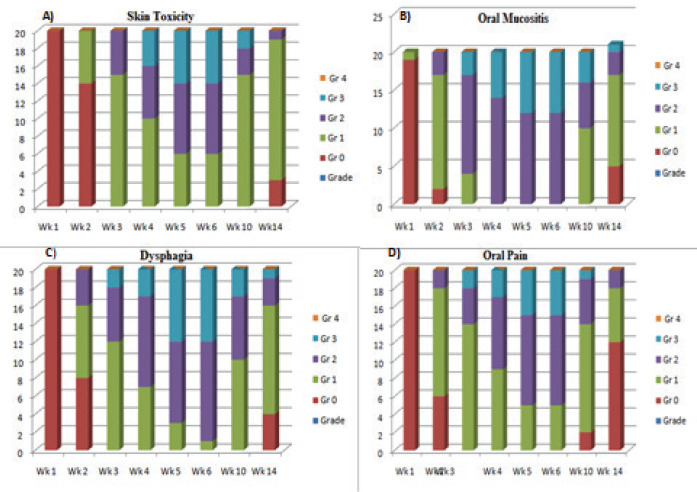
Stacked bars illustrating the temporal progression of acute toxicities for (a): Skin toxicity, (b): OM, (c): Pharynx/esophageal toxicity and (d): Oral pain.

Figure 3 demonstrates the weekly progression of acute toxicities during the treatment. On weekly analysis, Grade 2 OM was observed in 12 patients (60%) by the 6th week, with Grade 3 confluent OM appearing in the third week. Number of patients with Grade 3 or higher toxicities increased as treatment progressed, reaching 8 patients (40%) by the end of the 6th week, with no Grade 4 toxicity.

Grade 2 skin toxicities were observed in 8 patients (40%) and Grade 3 in 6 patients (30%) by the end of 6th week. Grade 2 and 3 dysphagia started appearing during the 2nd and 3rd weeks of treatment, with Grade 3 seen in 8 patients (40%) by the end of 6^th^ week and 11 patients (55%) had clinically Grade 2 toxicities with altered diet.

Grade 2 oral pains started appearing during the 2nd and 3rd weeks of treatment, while Grade 3 oral pain began in the 3rd and 4th weeks. By the end of treatment, Grade 3 toxicity was observed in 5 patients (25%) at the 6th week and Grade 2 toxicities were present in 10 patients (50%) at the 6th week.

Overall 8 patients (40%) had Grade 3 OM and odynophagia each, while 6 patients (30%) had Grade 3 skin toxicity and 5 patients (25%) had oral pain, respectively, with no Grade 4 toxicities were observed.

Two patients (10%) developed chemo-RT-induced Grade 2 anemia toxicities and 2 patients (10%) had Grade 2 neutropenia, with no patients experiencing thrombocytopenia. Overall toxicity evaluation 90 days after completion of RT showed OM and odynophagia in 1 patient (5%), with no skin toxicity or oral pain.

The median weight loss was 3 kg (range 1–5 kg), with 9 patients (45%) receiving IV fluids and 10 patients (50%) requiring tube feeding during RT. The median duration of enteral feeding was 20 days (range 12–40 days), with the median initiation time being 20 days (range 16–28 days) from the start of RT. Most patients were able to restart oral feeds within 5 weeks of completing RT.

**Figure 4.**
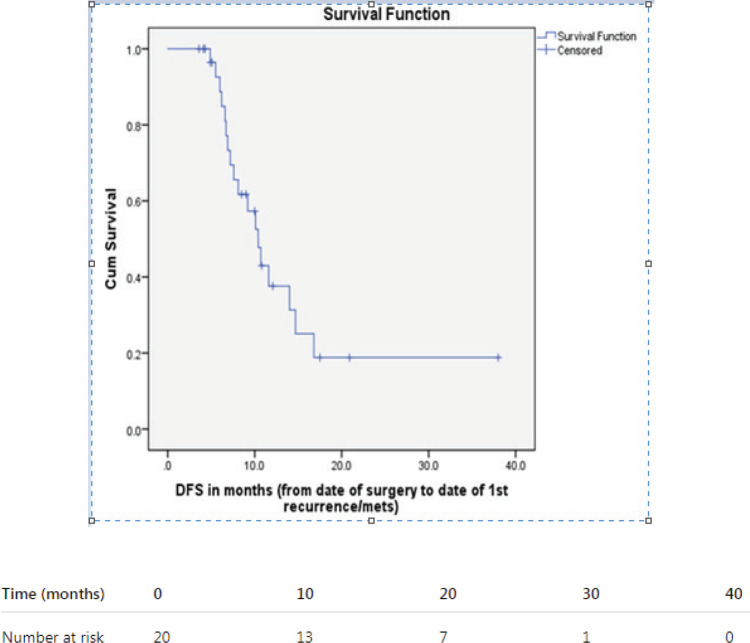
DFS curve.

**Figure 5.**
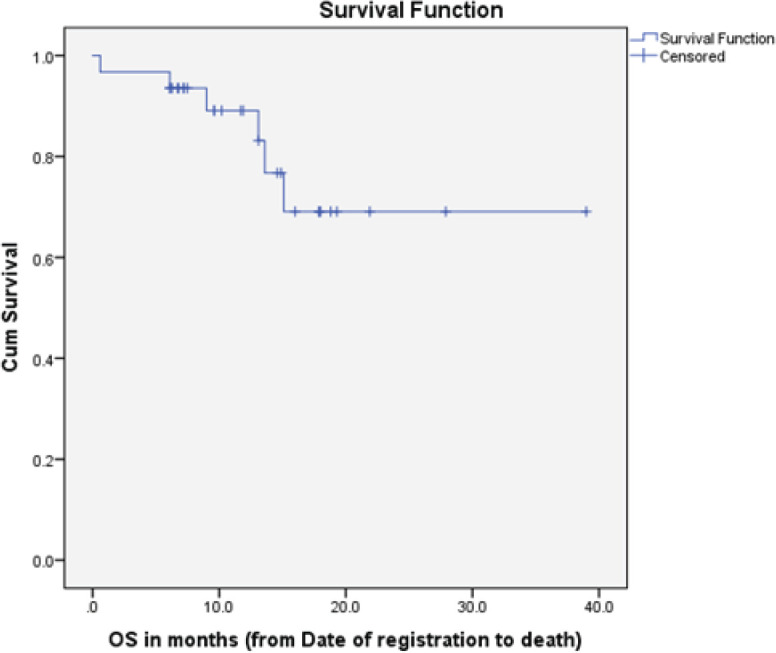
OS curve.

On clinical assessment at 1st month follow up 19 patients were without recurrence and one patient died due to covid pneumonitis. LRC at three month was 73.68% (14/19) with 4 local and 1 locoregional failures only. Five were given Palliative Chemotherapy. LRC at 1 year was 36.8% (7/19) with 8 local recurrences alone and 4 patients with distant metastasis were observed, 3 distant metastasis patients with loco and/or regional relapse and 1 patient had only distant metastasis. Figure 4 depicts Kaplan-Meier curve with Median disease free survival (DFS) of 10.4 month, however Median OS was not reached; four patients died: 2 due to primary disease, 1 due to distant metastasis in lung with loco-regional disease and 1 death related to other cause (Covid pneumonitis). Figure 5 depicts Kaplan-Meier curve of OS. The 1-year DFS rate and OS rate was **53%** (95% CI: 34%–83%) and **74% (95% CI: 56%–96%).** At 3 months, LRC was better in patients with low ITF disease compared to those with mid ITF involvement (85.7% versus 40%, *p* = 0.04); at one year, the difference narrowed (42.8% versus 40%, *p* = 0.50).

## Discussion

T4b OCSCC with ITF involvement treatment remains complex due to challenging anatomy and limited resectability. Recent evidence supports curative surgery with ITF clearance followed by adjuvant chemoradiotherapy. Our prospective study focused on such patients with low and mid ITF involvement and demonstrated that this multimodality approach is feasible, tolerable and may offer improved short-term control.

In our study, adjuvant CCRT was administered to all patients irrespective of margin or extranodal extension (ENE) status, as ITF involvement itself was considered a high-risk feature in line with our institutional practice. Few studies historically showed poor outcomes of T4b OCSCC with MS or ITF spread, where surgery alone is not sufficient to achieve good outcome. Prior reports, including those by Trivedi *et al* [24] and Katna *et al* [30], have similarly depicted the aggressive biology of ITF-involved tumours and warrant for intensified adjuvant therapy**.**

We observed a 3-month LRC rate of 73.7% and 1-year LRC of 36.8%, with a median DFS of 10.4 months. These outcomes are comparable to previously reported prospective and retrospective studies. For example Katna *et al* [30] Also reported acceptable disease control following compartmental resections with adjuvant therapy. Our findings align with Liao *et al*) [15], who showed better outcomes in infra-notch T4b OCSCC compared to more extensive disease. Similarly, Manish Mair *et al* [37] emphasized the benefit of adjuvant CCRT, particularly in improving DFS for advanced cases thus supporting our choice of intensified postoperative therapy as shown in Table 3.

Importantly, patients with low ITF involvement had better short-term outcomes than those with mid ITF involvement (3-month LRC: 85.7% versus 40%, *p* = 0.04) though this advantage narrowed at 1 year (42.8% versus 40%, *p* = 0.50) was not statistically significant, indicating that the short-term benefit seen with low ITF involvement does not persist over longer follow-up.

This supports ITF sub-stratification as a useful prognostic marker, as echoed by recent compartment-based approaches like those described by Liao *et al* [15], who reported better outcomes in infra-notch tumours and recently by Mahajan *et al* [16]**.** In cases of T4b oral cavity cancer, the outcomes of CCRT are not very promising; therefore, an initial approach involving ITF clearance followed by adjuvant CCRT may be considered a reasonable treatment option for these patients [35]**.**

Previous studies have shown that early invasion of the retroantral fat pad indicates posterior spread from buccal or alveolar primaries into the pterygoid muscles. Similarly, involvement of the masseter and medial pterygoid muscles represents involvement into the MS, which **is** linked to poorer outcomes. Trivedi *et al* [14, 22, 24, 25] and Katna *et al* [30] both reported that MS invasion has difficulty in margin clearance lead to poorer DFS, even with aggressive surgical approaches. In our study, standardized ITF clearance was designed to remove these high-risk areas and limit microscopic spread. However, the poorer DFS seen in patients with mid-ITF disease supports earlier findings that such extensions carry a worse prognosis.

Treatment compliance was excellent, and all patients completed planned therapy with integral supportive care strategies such as nutritional supplementation and oral prophylaxis. Grade 3 mucositis and odynophagia were seen in 40% of cases, with no Grade 4 toxicities reported. We have compare outcomes, toxicities were similar to studies by Dewan *et al* [36] and Mair *et al* [37], reported acceptable levels of Grade 3 toxicities with no unmanageable adverse effects indicating that CCRT using weekly cisplatin and 3D conformal RT is both feasible and tolerable. Most patients resumed oral intake within 5 weeks, suggesting acceptable functional outcomes post-treatment. The results of such similar studies are tabulated in Table 2.

Our study’s strength lies in its prospective nature, uniform protocol and anatomical stratification, providing focused insight into a rare but operable subgroup of T4b OCSCC. All patients were treated with standardized protocols and timely initiation of RT. Benefit of weekly toxicity monitoring and uniform follow-up added strength to the data quality.

However, certain limitations are the small sample size which limits the generalizability of findings and short follow-up duration is insufficient to draw definitive conclusions about long-term survival or recurrence patterns. Selection bias may be present due to variation in ITF involvement and patient co-morbidities, although strict inclusion criteria were aimed to reduce it. Moreover patients with similar staging who were unfit for surgery or received non-surgical management were not included as part of this prospective study protocol, so prevents our ability to attribute outcomes solely to the intervention. Future prospective studies including all cT4b patients treated during the study period should include comparative arm of surgery alone or non-surgical approaches to provide more generalizable and unbiased conclusions.

Although our study provides important insights into the feasibility and short-term outcomes of surgery with ITF clearance followed by adjuvant CCRT in selected T4b OCSCC patients, these results should be interpreted as early prospective observations. Given the aggressive biology of T4b disease and the potential for late recurrences, these findings must be regarded as preliminary rather than definitive, highlight the importance of extended follow-up and validation in larger multicentre studies, ideally using advanced RT techniques, integrating advanced imaging and biological markers. Optimization of treatment based on ITF compartment involvement may guide to improve result.

## Conclusion

In conclusion, this prospective study supports the use of surgery with ITF clearance followed by adjuvant CCRT as a viable treatment approach for selected patients with advanced OCSCC involving the low and mid ITF. The approach is not only feasible but also associated with encouraging early outcomes and manageable toxicity. These results are early observations and not definitive. Because of the aggressive nature of T4b disease and possible late recurrences, longer follow-up and larger studies are needed to validate these findings.

## Conflicts of interest

The authors have no conflicts of interest to declare.

## Funding

We also did not receive any funding for the present study from any source.

## Informed consent

Written informed consent was taken from all patients prior to the enrolment.

## Author contributions

Conceptualization: PM, RH, AKG, MR; Investigation and methodology: PM, RH, AKG, MR, RK, SPM, AKS, AB, AS, AA; Supervision: RH, MR, AKG, RK, SPM, AKS, AB; Data curation: PM, MR, AKG, RK, RH, SPM, AKS, AB, AP, VKM, RP, AM, VG, BPV; Analysis and interpretation: RH, MR, AKG, RK, SPM, AKS, AB, AP, VKM, SM; Writing of the original manuscript: PM, RH, AKG, MR; Writing of the review and editing: PM, RH, AKG, MR. All authors have proofread and approved the final version. All authors have contributed significantly to the article. All authors are in agreement with the concept of the manuscript.

**Permissions:** Approval by Institutional Ethics Committee (IEC No.166/20/Res Cell /RMLIMS/ 2020). The authors acknowledge that the full article has not been published in any journal nor it is under review in any other journal for publication.

**Supporting information**: Additional supporting information may be found online in the Supporting Information section at the end of the article.

## Figures and Tables

**Table 1.** Description of CTVs taken for different sites of oral cavity.

Tumor site	Pathological stage	CTV_primary	CTV_nodal
Buccal mucosa	T 1, T 2, T 3 N 0 T4N0 Any T N +	Postoperative tumor bed	Ipsilateral level IB-III (± IV) Ipsilateral level IB-IV ± Contralateral level I-IV LNs Ipsilateral level IB-IV ± Contralateral level I-IV LNs
Tongue	T 1, T 2, T 3 N 0 T4N0 Any T N +	Postoperative tumor bed	Bilateral level I-IV Level V when indicated
Alveolus/GBS	T 1-3 N0 T4 N0 Any T N +	Postoperative tumor bed	Ipsilateral level IB-III (± IV) Ipsilateral level IB-IV ± Contralateral level I-IV LNs Ipsilateral level IB-IV ± Contralateral level I-IV LNs
RMT	T 1-2 N0 T 3-4 N0/+	Postoperative tumor bed	Ipsilateral level IB-III (± IV) Bilateral level I-IV

Abbreviations: CTV- Clinical target volume, GBS- Gingiva-buccal sulcus, RMT-Retromolar trigone

**Table 2.** Patient and tumour characteristics.

Patient characteristics (*n* = 20)	No of patients (%)
Median age (Range)	39 years (32–67 years)
Male: Female	19:1
Tumor site Buccal mucosa Upper alveolus	19(95%) 1(5%)
cN stage cN0:N1:N2a:N2b:N2c	04 (20%):05(25%):06 (30%):04(20%): 01(05%)
Clinical ITF extent of involvement Low Mid	15 (75%) 05 (25%)
Differentiation WD MD PD	04 (20%) 15 (75%) 1 (05%)
DOI (in mm) ≥10 mm <10 mm	17 (85%) 03 (15%)
LVI:PNI – positive Bone involved ENE positive Margin-Close (1–5 mm): Involved (<1 mm)	11 (55%):12 (60%) 07 (35%) 05 (25%) 02 (10%): 01 (5%)
Pathological nodal stage (pN) pN0:pN1:pN2a:pN2b:pN3c	05 (25%):04 (20%):04 (20%):06 (30%): 01 (05%)
KPS 90:80	16 (80%):4 (20%)

Abbreviations: ITF: Infra Temporal Fossa, DOI: Depth of invasion, LVI: Lymphovascular invasion, PNI: Perineural invasion, ENE: Extranodal extension, KPS: **Karnofsky Performance Status, WD: Well differentiated, MD: Moderate differentiated, PD: Poorly Differentiated**

**Table 3.** Showing characteristics and outcomes from comparative studies.

Study	Type	Patient No.	Disease/group	Median FU (months)	Treatment	RT timing	RT technique	RT Dose/Chemo	LRC	DFS/DSS/PFS	OS	Key results/Conclusion
[22]	Retrospective	103 (T4a: 45, T4b: 58)	OCSCC T4a versus T4b	60	Surgery or Surgery + RT/CCRT (*n* = 63)	4–8 weeks post-op	3D conformal RT	60–66 Gy/ 30–33 fx; Chemo: weekly cisplatin 30 mg/m^2^ or biweekly with tegafur + leucovorin	LC: 71% versus 69%; Neck: 87% versus 74%	DFS: 66% versus 57%; DSS: 65% versus 60%	45% versus 49%	No significant difference between T4a and T4b. ≥2 positive nodes predicted poorer survival.
[15]	Retrospective	45	T4b OCSCC (Supra-notch: 7, Infra-notch: 38)	63	Surgery + RT ± CCRT	4–8 weeks post-op	3D conformal RT	60–66 Gy/ 30–33 fx; Chemo: cisplatin weekly or biweekly with tegafur + leucovorin	LC: 42.9% versus 74%; (*p* = 0.0254) Neck: 21.4% versus 83.8%), (*p* = 0.0001)	DFS: 14.3% versus 64.7% (*p* = 0.0004)	14.3% versus 55.3%	Infra-notch disease with no nerve invasion and pN0–1 had better local control. Perineural spread are critical determinants of prognosis
[24]	Prospective	30	OCSCC T4b	23	Radical surgery + RT (*n* = 26) or CCRT (*n* = 4)	Data not available	Data not available	Data not available	25 patients disease-free; 2 local recurrences	Not reported	1 disease-related death, 2 unrelated	Compartmental approach improved margin control without added morbidity.
[38]	Retrospective	110	OCSCC T4b		NACT + Surgery (*n* = 34), Non-surgical: CCRT (30), RT (2), Palliative RT (19)	Data not available	Data not available	NACT: 1–2 cycles DCF or other	Local failure: 82.8% (53/64)	PFS: 5.07 months	Surgery: 18 months; Non-surgery: 6.5 months	NACT improved resectability. Surgery offered better OS.
[36]	Retrospective	203	OCSCC with superior GBS involvement	15	ITF clearance (*n* = 56) versus No clearance (*n* = 157); RT ± CT	Data not available	Data not available	Pre-op RT: 50–54 Gy; Adjuvant RT: 60–66 Gy; pITF+: 60 Gy	Relapse: 17/39 (ITF), 42/105 (non-ITF)	DFS: ITF 58% versus non-ITF 49%	OS: ITF 40% versus non-ITF 36%	ITF clearance improved DFS and OS. Value of aggressive resection in selected patients.
[23]	Retrospective	73	OCSCC invading MS	Not reported	Surgery with ITF clearance	Data not available	Data not available	Data not available	LC: 69.4%	DFS: 58.3%	OS: N+: 42.6%, *N*–: 71.7%	ITF clearance effective in absence of nodal metastasis. Node positivity influenced survival.
[37]	Prospective	210 (T4a: 135, T4b: 75)	OCSCC	22–24	Surgery + RT (130), CCRT (80)	Data not available	Data not available	Data not available	LC: 49.6% (T4a) versus 41.1% (T4b)	DFS: 65.3% versus 42%; CCRT DFS: 41.6% versus 33.6% was 72.2% and 42.1%	OS: 49.6% versus 41.1% (p-0.518)	Local control comparable between both groups; CCRT benefit suggested in T4b cases, reinforcing its role in high-risk settings.
[39]	Retrospective	52	OCSCC T4b	24	ITF clearance + RT (36), CCRT (16); NACT (20)	Data not available	Data not available	Data not available	Local recurrence in ITF: 14 cases	Not reported	OS: 60%; 8 deaths due to disease	Close margins, ECE, posterior ITF, and supra-notch disease associated with higher recurrence rates, thus guide the extent of surgery and need for adjuvant therapy.
[30]	Retrospective	54	OCSCC T4b	29	Surgery (46) with ITF clearance; NACT (8) + Surgery; RT ± CT	Data not available	Data not available	RT: 60 Gy (range: 54–66 Gy); 3–6 cycles of cisplatin	2-year LRC: 52%; Recurrence in ITF: 7	DFS: 54%	OS: 54%	PNI, pT-stage, nodal positivity, and ITF invasion linked with poor outcomes.
[16]	Retrospective	154	OCSCC T4b: Low ITF (79), High ITF (63)	Not reported	NACT + Surgery (61) ± RT/CT/Def/Pall Care		IMRT	Data not available	Not reported	PFS similar in compartments ½ (*p* = 0.692); poor in 3 (*p* = 0.033)	Not reported	High-level (compartment 3c) invasion had the worst progression-free survival.Importance of detailed imaging-based compartmentalization to tailor treatment intensity and improve prognostic accuracy
Experimental Study, 2022	Prospective	20	T4 OCSCC with Mid & Low ITF	11.4	ITF clearance + Adjuvant CCRT	4–8 weeks post-op	3D conformal RT	Adjuvant 60–66 Gy/30–33 fx; 5–7 cycles of cisplatin	LRC @ 3 months: 73.7%; @ 1 year: 36.8%	Median DFS: 10.4 months	Median OS: Not reached	Better LRC in low ITF versus mid ITF: 85.7% versus 40% at 3 months (*p* = 0.04)

Abbreviations: **OCSCC**: Oral cavity squamous cell carcinoma, **RT**: Radiotherapy, **CCRT**: Concurrent chemoradiotherapy, **LC/LRC**: Local (or loco-regional) control, **DFS/DSS/PFS**: Disease-free / Disease-specific / Progression-free survival, **OS**: Overall survival, **fx**: Fractions, **ITF**: Infratemporal fossa, **NACT**: Neoadjuvant chemotherapy, **GBS**: Gingivobuccal sulcus, **PNI**: Perineural invasion, **p-ITF+** : Pathologic ITF positive, **ECE**: Extra-capsular extension
